# Improving antifungal stewardship in dermatology in an era of emerging dermatophyte resistance

**DOI:** 10.1016/j.jdin.2024.01.004

**Published:** 2024-01-19

**Authors:** Avrom S. Caplan, Jeremy A.W. Gold, Dallas J. Smith, Shari R. Lipner, Peter G. Pappas, Boni Elewski

**Affiliations:** a The Ronald O. Perelman Department of Dermatology, NYU Grossman School of Medicine, New York, New York; b Mycotic Diseases Branch, Centers for Disease Control and Prevention, Atlanta, Georgia; c Department of Dermatology, Weill Cornell Medicine, New York, New York; d Division of Infectious Diseases, University of Alabama at Birmingham, Birmingham, Alabama; e Department of Dermatology, University of Alabama at Birmingham, Birmingham, Alabama

**Keywords:** antifungal resistance, dermatophyte, infection, Trichophyton indotineae, Trichophyton mentagrophytes, Trichophyton rubrum, tinea, ringworm

*To the Editor:* Dermatophyte infections are the most common mycosis worldwide, affecting an estimated 25% of the world’s population.^1^ Recently, antimicrobial-resistant strains of the dermatophytes *T. rubrum* and *T. indotineae* have spread globally, causing outbreaks of extensive, difficult-to-treat infections in South Asia. *T. indotineae* is now detected in 11 US states.^2^ These infections generally do not improve with topical antifungals alone or oral terbinafine unlike typical dermatophytoses.^2^^,^^3^ The precise factor(s) underlying the emergence of resistance in dermatophytes is unclear. In South Asia, inappropriate use of topical combination antifungal and corticosteroid products is temporally associated with the emergence of resistant dermatophytes^3^, and improper use of antifungal medications has elsewhere been associated with antifungal resistance.^4^ Thus, urgent attention to antifungal stewardship (AFS) is warranted. As experts in skin, hair, and nail infections, dermatologists are well-poised to address the emerging threat of antimicrobial-resistant dermatophytoses and implement AFS programs.

A core component of AFS is confirming suspected fungal infections. Clinical mimickers of dermatophytoses are common, and laboratory confirmation can help ensure the correct diagnosis. Potassium hydroxide preparations or histopathology using periodic acid-Schiff can confirm the presence of hyphae; fungal cultures or polymerase chain reaction methods identify causative species. Certain polymerase chain reaction assays can identify squalene epoxidase mutations associated with decreased terbinafine susceptibility; advanced molecular techniques are required to identify the novel, frequently antimicrobial-resistant organism *T indotineae*.^3^

Unfortunately, substantial barriers hamper the widespread adoption of testing for suspected dermatophytoses. In-office testing is restricted by Clinical Laboratory Improvement Amendments (CLIA) regulations, and dermatologists may lack time to perform potassium hydroxide preparations. Cultures may take weeks to result, and lack of access to specialized laboratories may limit species identification and resistance testing. Low reimbursement rates for potassium hydroxide and lack of reimbursement for other tests and mycological sampling can also hinder testing. Testing may not be feasible before therapy is initiated or may require repetition and clinical interpretation to reduce false positive or confirm negative results.

Additional barriers may arise when treating dermatophytoses, especially where first-line topical antifungal medications prove ineffective. In such instances, dermatologists should evaluate adherence to therapy, alternate diagnoses, and possibly, antifungal resistance. Resistant infections may require treatment with itraconazole. However, dermatologists may be unfamiliar with this medication, which has variable drug absorption, drug interactions, and serious potential side effects requiring monitoring. Insurance may deny or require previous authorizations for these therapies, creating additional obstacles.

Successful AFS efforts hinge on addressing these barriers by decreasing regulatory and insurance obstacles that limit access to testing and second-line therapies. AFS programs can also emphasize improved laboratory capacity and continuing education programs that teach bedside diagnostics and advanced therapeutics. Focus on patient-reported outcome measures^5^ could be incorporated into AFS programs, in addition to clinical and mycologic cure as points of evaluation. Updated guidelines are necessary to guide diagnostics and therapy in the era of emerging antifungal resistance. Expanded research could elucidate resistant dermatophyte epidemiology, virulence, and pathogenesis. Addressing resistant dermatophytosis is a critical emerging challenge. Dermatologists are well positioned to implement AFS into daily practice and help ensure the best outcomes for patients.

## Conflicts of interest

None disclosed.

## References

[1] Havlickova B., Czaika V.A., Friedrich M. (2008). Epidemiological trends in skin mycoses worldwide. Mycoses.

[2] Cañete-Gibas C.F., Mele J., Patterson H.P. (2023). Terbinafine-resistant dermatophytes and the presence of trichophyton indotineae in North America. J Clin Microbiol.

[3] Uhrlaß S., Verma S.B., Gräser Y. (2022). Trichophyton indotineae-An emerging pathogen causing recalcitrant dermatophytoses in India and worldwide-a multidimensional perspective. J Fungi (Basel).

[4] Srinivasan A., Lopez-Ribot J.L., Ramasubramanian A.K. (2014). Overcoming antifungal resistance. Drug Discov Today Technol.

[5] Snyder A.M., Chen S.C., Chren M.M. (2023). Patient-reported outcome measures and their clinical applications in dermatology. Am J Clin Dermatol.

